# Clinical significance of p53 protein expression and *TP53* variation status in colorectal cancer

**DOI:** 10.1186/s12885-022-10039-y

**Published:** 2022-08-31

**Authors:** Kyoung Min Kim, Ae-Ri Ahn, Ho Sung Park, Kyu Yun Jang, Woo Sung Moon, Myoung Jae Kang, Gi Won Ha, Min Ro Lee, Myoung Ja Chung

**Affiliations:** 1 Departments of Pathology, Jeonbuk National University Medical School, Research Institute of Clinical Medicine of Jeonbuk National University, Biomedical Research Institute of Jeonbuk National University Hospital, and Research Institute for Endocrine Sciences, San 2-20 Keumam-Dong Dukjin-gu, Jeonju, 54907 Republic of Korea; 2 Department of Surgery, Jeonbuk National University Medical School, Research Institute of Clinical Medicine of Jeonbuk National University, and Biomedical Research Institute of Jeonbuk National University Hospital, Jeonju, Republic of Korea

**Keywords:** Colorectal cancer, TP53, Next-generation sequencing, Immunohistochemistry

## Abstract

In human colorectal cancer (CRC), *TP53* is one of the most important driver genes. Immunohistochemistry (IHC) has been used most often to assess the variational status of *TP53*. Recently, next-generation sequencing (NGS) of the *TP53* gene has increased. However, to our knowledge, a comparison between *TP53* status evaluated by IHC and NGS has not been studied. Therefore, the primary aim of this study was to compare the clinical effect of *TP53* status evaluated by IHC and NGS in patients with CRC. The secondary aim was to investigate the correlation between expression of p53 by IHC and variational status of *TP53* by NGS. We performed immunohistochemical staining of p53 and sequencing of *TP53* by NGS in 204 human samples of CRC. We then analyzed the correlation between variational status of *TP53* and p53 expression, along with their prognostic impact in CRC patients. There was significant correlation between p53 expression and *TP53* variation, *TP53* variation and higher N stage, and positive p53 expression and higher N stage. Positive IHC expression of p53 was significantly associated with overall survival (OS) of CRC patients by univariate analysis and was revealed as an independent prognostic factor by multivariate analysis. Additionally, the nonsense/frameshift p53 expression pattern showed a significantly better prognosis than the wild type and missense p53 expression patterns. However, the variational status of *TP53 was not significant in OS of CRC patients.* These results suggest that IHC expression of p53 protein correlates with variation status of *TP53* and expression of p53 protein rather than variation status of *TP53* has more significant impact on the OS of CRC patients.

**Supplementary Information:**

The online version contains supplementary material available at 10.1186/s12885-022-10039-y.

## Introduction

Colorectal cancer (CRC) is the third most commonly diagnosed cancer and the second leading cause of cancer-related death worldwide [1]. In human CRCs, *TP53* along with *APC*, *KRAS*, and *SMAD4* are frequently mutated genes by genome-wide analysis [2, 3]. Variations of these genes are thought to contribute to the various properties of colon cancer cells, such as stemness, proliferation, dedifferentiation, impaired genome maintenance, invasiveness, and metastatic ability [4]. Among the genes that are frequently mutated, the variation of *TP53* gene is one of the key genetic steps in development of CRC [5].

The well-known tumor suppressor p53, which is the product of the *TP53* gene, induces cell-cycle arrest, senescence, or apoptosis under cellular stress, such as DNA damage, hypoxia, nutrient depletion, and oncogenic signaling [6, 7]. The p53 protein promotes these responses by regulating target molecules, including p21, Puma, Tiger, and PAI-1 [8]. *TP53* variations can be subdivided into missense variations and nonsense/frameshift variations. The *TP53* loss-of-function variation promotes tumorigenesis due to decreased p53 target induction under cellular stress [5]. Accumulation of evidence indicates that missense-type variations at the DNA binding domain of *TP53* can induce oncogenic function [9, 10].

CRC is reported to be the most common cancer entity that harbors *TP53* variation, with 43.28% of CRCs reported to have *TP53* variation (IARC *TP53* database, R20; https://p53.iarc.fr/TP53SomaticVariations.aspx, accessed on 27 October 2021). Therefore, the roles of alterations in *TP53* are actively studied in CRCs. *TP53* variations have been reported to be correlated with the poor prognosis of patients with CRC [11, 12]. In patients with advanced stage of CRC with metastasis, the rate of *TP53* variations is reported to be as high as 80% [13]. In addition, missense-type *TP53* variations are reported to be associated with chemoresistance in CRCs [14].

Immunohistochemical staining of p53 has long been used as a surrogate marker for variation status of *TP53*. However, because of the high yield in genes or genomic regions that can be evaluated by next-generation sequencing (NGS) at low cost and relatively faster turnaround time, sequencing of the *TP53* gene through NGS is increasing. Recently, there has been a study on the relationship between immunmohistochemical expression of p53 and *TP53* variation status in ovarian cancer [15]. *TP53* variations can be divided into missense and nonsense/frameshift variations [15]. Missense variations disturb MDM2-induced ubiquitination and degradation of p53, which lead to aberrant p53 accumulation in the nucleus [15]. Nonsense/frameshift variations cause premature stop codons and trigger nonsense-mediated RNA collapse, and protein translation can be disrupted by frameshifts or aberrant splicing [15]. A nonsense/frameshift variation in *TP53* can cause a decrease or complete absence of p53 protein expression [15]. However, the interpretation of p53 IHC varies and has not been confirmed in many cancers including CRC.

In this study, we used IHC to investigate the cutoff value of p53 expression that is highly relevant to survival of CRC patients and the optimal cutoff value reflecting *TP53* variation and compared the clinical significance of the two values. We do not believe that the optimal cutoff values reflecting variations must coincide with those that best reflect the pathological role of p53 expression in cancer. Therefore, we believe it is meaningful to find and compare cutoff values of p53 expression that are highly relevant to prognosis and cutoff values that reflect the status of variations. Furthermore, we aimed to compare the prognostic effects of p53 protein expression by IHC and *TP53* variation status by NGS in CRC.

## Materials and methods

### Patients and follow-up

In total, 204 patients with CRC who underwent surgery at Jeonbuk National University Hospital between May 2018 and May 2019 were enrolled in this study. Medical records were reviewed to obtain clinicopathologic information of sex, age, histologic grade, tumor location, tumor size, carcinoembryonic antigen (CEA), T stage, N stage, and TNM stage, as summarized in Table 5. For analysis, the entire colon was divided into the right and the left. The right-side colon was defined as the segment from the cecum to the proximal two-thirds of the transverse colon, and the left-side colon was defined as the segment from the distal one-third of the transverse colon to the rectum. Histologic slides were reviewed by two pathologists according to the WHO classification of tumors of the digestive system [16]. The TNM stage of the CRC patients was classified by the 8th edition of the American Joint Committee Cancer Staging System [17]. Postoperative surveillance for CRC patients was performed every 3 months. Laboratory tests including serum tumor marker CEA were performed. Abdominal computed tomography (CT) was used to detect recurrence and metastasis. This study was approved by the Institutional Review Board of Jeonbuk National University Hospital (IRB number, CUH 2019–04-053) and was conducted according to the Declaration of Helsinki.

### Next-generation sequencing (NGS)

Targeted NGS was performed using formalin-fixed paraffin-embedded (FFPE) tumor tissue. Hematoxylin and eosin-stained slides were reviewed, and tumor areas with sufficient viable tumor cells were marked and used as a guide for macrodissection. Areas with greater than 50% tumor volume were used for examination. In brief, total nucleic acid was isolated from tumor tissue using a RecoverAll Total Nucleic Acid Isolation Kit for FFPE (Ambion, Austin, TX, USA) according to the manufacturer’s specifications. After extracting DNA and RNA from FFPE specimens, library preparation for an Oncomine Comprehensive Assay v1 (OCAv1, Thermo Fisher Scientific, Waltham, MA, USA) was performed. An IonTorrent S5 XL platform was used for sequencing following the manufacturer’s instructions. The OCAv1 is an amplicon-based targeted assay and includes the entire coding sequence of exons 2–11 of *TP53*. Reads were aligned to the hg19 reference genome, and variants with allele frequencies less than 3% were excluded. The reference transcript for *TP53* analysis was NM_000546.5. Genomic data obtained by sequencing were analyzed by IonReporter Software v5.6 (Thermo Fisher Scientific). Additionally, a manual review of the variant call format file, integrated genomic viewer and various public databases was conducted. And the p53 missense variations that were not identified as pathogenic were excluded from the study.

### Immunohistochemistry (IHC)

**Fig. 1:**
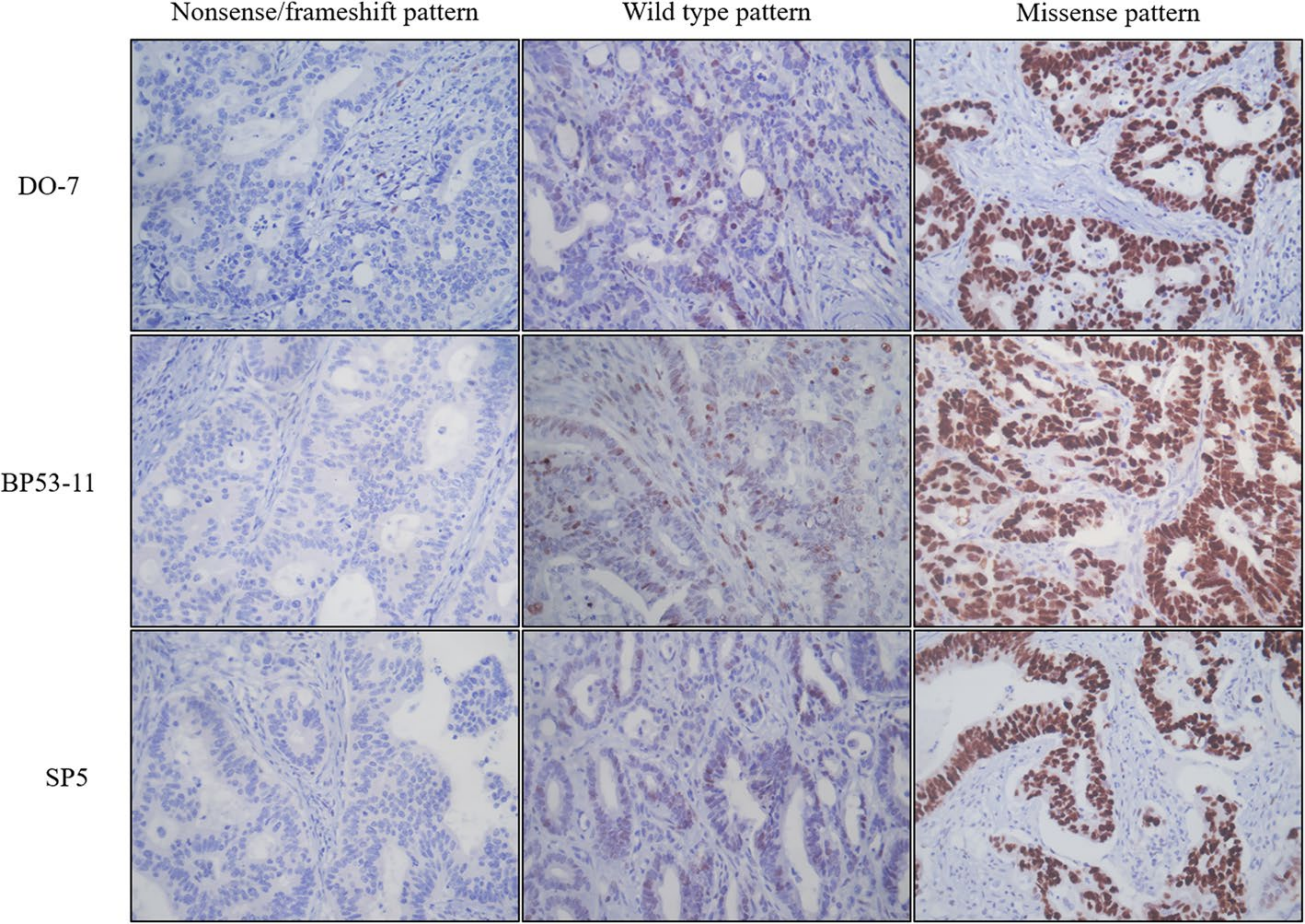
Immunohistochemical expression of p53 in colorectal carcinoma tissue. We subdivided the p53 expression into nonsense/frameshift, wild type, and missense type pattern. The nonsense/frameshift pattern showing no expression, wild type pattern showing focal nuclear expression of p53, and missense pattern showing diffuse strong nuclear expression of p53 (original magnification: × 400)

Immunohistochemical staining for p53 antibodies of DO-7 (dilution: ready to use, Roche Diagnostics, Mannheim, Germany), Bp53–11 (dilution: 1:100, Progen Biotechnik GmbH, Heidelberg, Germany), and SP5 (dilution: 1:100, Abcam, Cambridge, United Kingdom) was utilized in the present study. Clone DO-7 and Bp53–11 were targeted to bind to the N-terminal of p53 protein. However, the epitope of SP5 clone is not determined. Tissue sections were stained on a Benchmark ULTRA, automated immunohistochemistry stainer (Ventana Medical Systems Inc., Tucson, AZ, USA) using OptiView DAB IHC Detection Kit (Ventana Medical Systems Inc.), as following procedure. Heat induced epitope retrieval was performed with ULTRA cell conditioning solution (ULTRA CC1, Ventana Medical Systems) for 32 min at 100 °C. Optiview Peroxidase Inhibitor (3% Hydrogen peroxide solution) was incubated for 4 min and Primary Antibodies were incubated for 12 min at 37°, followed by OptiView DAB IHC Detection Kit (Optiview HQ Universal Linker 8 min, Optiview HRP Multimer 8 min, Optiview DAB&Optiview H2O2 8 min, Optiview Copper 4 min). OptiView HQ Universal Linker contains a cocktail of HQ-labeled (HQ is a proprietary hapten covalently attached to the goat antibodies) antibodies (goat anti-mouse IgG, goat anti-mouse IgM, and goat anti-rabbit) (< 50 μg/mL) in a buffer and OptiView HRP Multimer contains a mouse monoclonal anti-HQ-labeled HRP antibody (< 40 μg/mL) in a buffer. Then slides are removed from the stainer and counterstaining was obtained off-line using Mayer’s hematoxylin (ScyTek, UT, USA) manually. Staining was performed on the whole section of the representative slide and was evaluated by two pathologists (KMK and MJC) without knowledge of the clinical status of the patient. Nuclear staining was considered a positive reaction. Tumor cells were considered positive when they showed moderate to strong nuclear staining. The proportion of p53 positive cells was recorded semiquantitatively using 5% increments. Representative findings of p53 immunohistochemical staining for three clones are shown in Fig. 1. In previous reports, in a small number of cases, cytoplasmic staining of p53 IHC was reported. However, in the current study, cytoplasmic staining pattern have not been observed, and therefore was not considered when calculating the positive proportion.

We set two cut-off points for immunohistochemical expression of p53. To investigate the prognostic impact of p53 IHC expression in CRC patients, we performed receiver operating characteristic (ROC) curve analysis. The cut-off points were determined at the points with the highest area under the curve (AUC) to predict cancer related death of the patients. And the cut-off points that analyzed to best predict the cancer related death of the patients were 55, 50, and 30% for DO-7, Bp53–11, and SP5, respectively. Thereafter, CRC patients with p53 expression level equal to or less than cut-off points were classified as the negative expression group, and patients with greater than cut-off points were classified as the positive expression group.

Next, ROC curve analysis to set the cut-off points of the p53 IHC expression according to the *TP53* variational status was performed. The *TP53* variation was classified into two types (missense and nonsense/frameshift variations), and the cut-off values at the highest AUC to predict missense and nonsense/frameshift variations of *TP53* gene was set. The cut-off points for predicting missense variation were 80, 50, and 70% for DO-7, Bp53–11, and SP5, respectively. And cut-off point for predicting nonsense/frameshift variation was 1, 1, and 2% for DO-7, Bp53–11, SP5, respectively. In summary, CRCs with a p53 expression proportion between the two cut-off points were classified as wild-type expression patterns. And the other cases were classified as an aberrant expression type.

### Statistical analysis

The prognosis of CRC patients was evaluated for overall survival (OS) and relapse-free survival (RFS) through March 2021. In the OS analysis, death of the patient as a consequence of CRC was treated as an event. Patient death due to other causes or alive at the last follow-up were censored. In RFS analysis, relapse of CRC or patient death by CRC were treated as an event. Death of a patient due to other causes or alive at the last follow-up without relapse were censored. Cox proportional hazards regression analysis and Kaplan-Meier survival analysis were used to evaluate the prognosis of CRC patients. Pearson’s chi-square test was used to investigate the relationships between p53 expression and *TP53* variation status with other clinicopathological factors and the correlation between p53 expression and *TP53* variation. SPSS software (IBM, version 19.0, Armonk, NY) was used for statistical analysis. *P* values less than 0.05 were considered statistically significant.

## Results

### *TP53* variation analysis

We used targeted NGS (by OCAv1) to characterize CRC for *TP53* variations. For this method, *TP53* variation analysis was performed on the entire exome. The *TP53* variations were observed in 73% (149/204) and are summarized in Table 1, supplemental Tables 1 and 2. Of the *TP53* variations, 108 (72.5%) were missense variations (MS), 23 (15.4%) were nonsense variations (NS), and 18 (12.1%) were frameshift variations (FS). Among the functional domains of *TP53*, variations were observed most often in the DNA binding domain (DBD), in 86.6% of patients (129/149 cases). By variation type, 98.2% of MS variations (106/108), 43.5% of NS variations (10/23), and 72.2% of FS variations (13/18) were observed in DBD. Compared to MS or FS variations, NS variations were more commonly observed in domains other than the DBD, 30.4% (7/23) in the tetramerization domain and 26.1% (6/23) in the nuclear localization signaling (NLS) region. MS variations were evenly distributed in the subregions within the DBD (L2, L3, LSH, and other). However, NS and FS variations were observed mostly in non-zinc binding regions (excluding L2, L3, and LSH), at 91.3% (21/23) and 83.3% (15/18), respectively.

**Table 1:** Summary of TP53 variations for 204 colorectal carcinoma patients

	Total ***n*** = 149	Missense ***n*** = 108	Nonsense ***n*** = 23	Frameshift ***n*** = 18
**Functional domains**				
**Transactivation**	0	0	0	0
**Proline rich region**	3	0 (0%)	0 (0%)	3 (16.7%)
**DNA binding region**	129	106 (98.1%)	10 (43.5%)	13 (72.2%)
**Nuclear localization signalling**	6	0 (0%)	6 (26.1%)	0 (0%)
**Tetramerization**	9	1 (0.9%)	7 (30.4%)	1 (5.6%)
**Regulatory**	2	1 (0.9%)	0 (0%)	1 (5.6%)
**Sub-regions of DB domain**				
**L2**	31	28 (25.9%)	1 (4.3%)	2 (11.1%)
**L3**	22	20 (18.5%)	1 (4.3%)	1 (5.6%)
**LSH**	31	31 (28.7%)	0 (0%)	0 (0%)
**Other**	65	29 (26.9%)	21 (91.3%)	15 (83.3%)
**Exons**				
**Exon4**	9	1 (0.9%)	1 (4.3%)	7 (38.9%)
**Exon5**	36	33 (30.6%)	1 (4.3%)	2 (11.1%)
**Exon6**	22	12 (11.1%)	7 (30.4%)	3 (16.7%)
**Exon7**	26	24 (22.2%)	0 (0%)	2 (11.1%)
**Exon8**	44	35 (32.4%)	7 (30.4%)	2 (11.1%)
**Exon9**	1	0 (0%)	1 (4.3%)	0 (0%)
**Exon10**	10	2 (1.9%)	6 (26.1%)	1 (5.6%)
**Exon11**	2	1 (0.9%)	0 (0%)	1 (5.6%)

### Association between immunohistochemical p53 expression and *TP53* variation

Despite the increased incidence of NGS testing in CRCs, IHC is used most commonly to evaluate *TP53* status. Therefore, we investigated the correlation of immunohistochemical expression of p53 with *TP53* variations. In this study, we classified p53 expression based on two criteria. First, p53 expression was categorized as wild type pattern or aberrant type pattern, and this classification showed significant correlation with *TP53* variation (DO-7: *P* < 0.001, Bp53–11: *P* < 0.001, SP5: *P* < 0.001) (Table 2). Sensitivity and specificity for detecting TP53 variation using this criterion were summarized in Table 4. The other criterion of classifying p53 expression into positive and negative groups was also significantly correlated with *TP53* variation (DO-7: *P* < 0.001, Bp53–11: *P* < 0.001, SP5: *P* = 0.001) (Table 2). Sensitivity and specificity for detecting *TP53* variation using this criterion were listed in Table 4.

**Table 2:** Correlation of TP53 variation with two different cut-off points for immunohistochemical expression of p53

		p53 IHC (DO-7)		***p***	p53 IHC (DO-7)		***p***	p53 IHC (Bp53–11)		***p***	p53 IHC (Bp53–11)		***p***	p53 IHC (SP5)		***p***	p53 IHC (SP5)		***p***
		Wild type pattern (1 ~ 79%)	Aberrant type pattern (0% or ≥ 80%)		Negative (≤55%)	Positive (> 55%)		Wild type pattern (1 ~ 49%)	Aberrant type pattern (0% or > 50%)		Negative (≤50%)	Positive (> 50%)		Wild type pattern (2 ~ 69%)	Aberrant type pattern (≤1% or ≥ 70%)		Negative (≤30%)	Positive (> 30%)	
**All cases**	204	53 (26%)	151 (74%)		80 (39.2%)	124 (60.8%)		49 (24%)	155 (76%)		88 (43.1%)	116 (56.9%)		54 (26.5%)	150 (73.5%)		65 (31.9%)	139 (68.1%)	
***TP53*variation status**																			
**Wild type**	55	34 (61.8%)	21 (38.2%)		38 (69.1%)	17 (30.9%)		38 (69.1%)	17 (30.9%)		41 (74.5%)	14 (25.5%)		42 (76.4%)	13 (23.6%)		27 (49.1%)	28 (50.9%)	
**Mutant type**	149	19 (12.8%)	130 (87.2%)	< 0.001	42 (28.2%)	107 (71.8%)	< 0.001	11 (7.4%)	138 (92.6%)	< 0.001	47 (31.5%)	102 (68.5%)	< 0.001	12 (8.1%)	137 (91.9%)	< 0.001	38 (25.5%)	111 (74.5%)	0.001

As mentioned above, *TP53* variation can be further classified into missense and nonsense/frameshift types. Accordingly, we subdivided the p53 aberrant type pattern into missense and nonsense/frameshift type. This subgrouping of p53 expression showed a significant correlation with *TP53* variation types (Table 3). The sensitivity, specificity, and accuracy for detecting *TP53* variations are shown in Table 4.

**Table 3:** Correlation between p53 immunohistochemical expression pattern and TP53 variation type

	Total	p53 IHC (DO-7)			***p***	p53 IHC (Bp53–11)			***p***	p53 IHC (SP5)			***p***
		Wild type pattern (1 ~ 79%)	Aberrant type pattern			Wild type pattern (1 ~ 49%)	Aberrant type pattern			Wild type pattern (2 ~ 69%)	Aberrant type pattern		
			Nonsense/frameshift pattern (0%)	Missense pattern (≥80%)			Nonsense/frameshift pattern (0%)	Missense pattern (> 50%)			Nonsense/frameshift pattern (≤1%)	Missense pattern (≥70%)	
**All cases**	204	53 (26%)	40 (19.6%)	111 (54.4%)		49 (24%)	39 (19.1%)	116 (56.9%)		54 (26.5%)	45 (22.1%)	105 (51.5%)	
***TP53*variation status**													
**Wild type**	55	34 (61.8%)	9 (16.7%)	11 (20.4%)		38 (70.4%)	2 (3.7%)	14 (25.9%)		42 (77.8%)	7 (13%)	5 (9.3%)	
**Missense variation**	108	12 (11.1%)	2 (1.8%)	95 (87.2%)		7 (6.4%)	2 (1.8%)	100 (91.7%)		8 (7.3%)	2 (1.8%)	99 (90.8%)	
**Nonsense/frameshift variation**	41	7 (17.1%)	29 (70.7%)	5 (12.2%)	< 0.001	4 (9.8%)	35 (85.4%)	2 (4.9%)	< 0.001	4 (9.8%)	36 (87.8%)	1 (2.4%)	< 0.001

**Table 4:** Sensitivity, specificity, and accuracy of p53 immunohistochemistry for detecting *TP53* variation

Variation type	Sensitivity	Specificity	Accuracy
p53 (DO-7)			
Binary (IHC: wild/aberrant)	87.2%	61.8%	80.4%
Binary (IHC: positive/negative)	71.8%	69.1%	71.1%
Missense variation	87.2%	82.3%	85.3%
Nonsense/frameshift variation	70.7%	93.3%	88.7%
Wild type	61.8%	87.9%	80.9%
p53 (Bp53–11)			
Binary (IHC: wild/aberrant)	92.6%	69.1%	86.3%
Binary (IHC: positive/negative)	68.5%	74.5%	70.1%
Missense variation	91.7%	82.3%	87.7%
Nonsense/frameshift variation	85.4%	97.5%	95.1%
Wild type	70.4%	93.3%	86.8%
p53 (SP5)			
Binary (IHC: wild/aberrant)	91.9%	76.4%	87.7%
Binary (IHC: positive/negative)	74.5%	49.1%	67.6%
Missense variation	90.8%	92.7%	92.2%
Nonsense/frameshift variation	87.8%	94.5%	93.1%
Wild type	77.8%	92.6%	88.2%

### Immunohistochemical expression of p53 and *TP53* variation in CRC patients and their association with clinicopathologic characteristics

Association between the clinicopathologic factors of *TP53* variation and p53 expression is summarized in Table 5. The aberrant p53 (DO-7) expression pattern was significantly associated with lower histologic grade, higher N stage, and TNM stage. The positive p53 (DO-7) expression group showed a significant association with left-side CRC, higher N stage, and TNM stage. The aberrant p53 (Bp53–11) expression pattern was significantly associated with higher N stage, and TNM stage. The positive p53 (DO-7) expression group showed a significant association with left-side CRC. The aberrant p53 (SP5) expression pattern was significantly associated with smaller tumor size, higher N stage, and TNM stage. The *TP53* variation showed a significant correlation with smaller tumor size, higher N stage, and TNM stage.

**Table 5:** Association between clinicopathologic factors to *TP53* variation and p53 IHC expressions

Characteristics	Total	***TP53*** variation		***p***	p53 IHC (DO-7)		***p***	p53 IHC (DO-7)		***p***	p53 IHC (Bp53–11)		***p***	p53 IHC (Bp53–11)		***p***	p53 IHC (SP5)		***p***	p53 IHC (SP5)		***p***
		Wild	Variation		Wild type pattern (1 ~ 79%)	Aberrant type pattern (0% or ≥ 80%)		Negative (≤55%)	Positive (> 55%)		Wild type pattern (1 ~ 49%)	Aberrant type pattern (0% or > 50%)		Negative (≤50%)	Positive (> 50%)		Wild type pattern (2 ~ 69%)	Aberrant type pattern (≤1% or ≥ 70%)		Negative (≤30%)	Positive (> 30%)	
**All cases**	204	55 (27%)	149 (73%)		53 (26%)	151 (74%)		80 (39.2%)	124 (60.8%)		49 (24%)	155 (76%)		88 (43.1%)	116 (56.9%)		54 (26.5%)	150 (73.5%)		65 (31.9%)	139 (68.1%)	
**Sex**																						
**Male**	117	35 (29.9%)	82 (70.1%)		34 (29.1%)	83 (70.9%)		50 (42.7%)	67 (57.3%)		32 (27.4%)	85 (72.6%)		53 (45.3%)	64 (54.7%)		34 (29.1%)	83 (70.9%)		40 (34.2%)	77 (65.8%)	
**Female**	87	20 (23%)	67 (77%)	0.27	19 (21.8%)	68 (78.2%)	0.245	30 (34.5%)	57 (65.5%)	0.232	17 (19.5%)	70 (80.5%)	0.197	35 (40.2%)	52 (59.8%)	0.47	20 (23%)	67 (77%)	0.331	25 (28.7%)	62 (71.3%)	0.408
**Age (years)**																						
**< 50**	12	4 (33.3%)	8 (66.7%)		2 (16.7%)	10 (83.3%)		5 (41.7%)	7 (58.3%)		3 (25%)	9 (75%)		5 (41.7%)	7 (58.3%)		2 (16.7%)	10 (83.3%)		4 (33.3%)	8 (66.7%)	
**≥ 50**	192	51 (26.6%)	141 (73.4%)	0.608	51 (26.6%)	141 (73.4%)	0.448	75 (39.1%)	117 (60.9%)	0.858	46 (24%)	146 (76%)	0.935	83 (43.2%)	109 (56.8%)	0.916	52 (27.1%)	140 (72.9%)	0.427	61 (31.8%)	131 (68.2%)	0.91
**Histologic grade**																						
**Well or Moderate**	167	42 (25.1%)	125 (74.9%)		38 (22.8%)	129 (77.2%)		64 (38.3%)	103 (61.7%)		36 (21.6%)	131 (78.4%)		72 (43.1%)	95 (56.9%)		42 (25.1%)	125 (74.9%)		53 (31.7%)	114 (68.3%)	
**Poor**	37	13 (35.1%)	24 (64.9%)	0.216	15 (40.5%)	22 (59.5%)	0.026	16 (43.2%)	21 (56.8%)	0.579	13 (35.1%)	24 (64.9%)	0.08	16 (43.2%)	21 (56.8%)	0.989	12 (32.4%)	25 (67.6%)	0.364	12 (32.4%)	25 (67.6%)	0.934
**Site**																						
**Right side**	69	19 (27.5%)	50 (72.5%)		23 (33.3%)	46 (66.7%)		34 (49.3%)	35 (50.7%)		19 (27.5%)	50 (72.5%)		37 (53.6%)	32 (46.4%)		21 (30.4%)	48 (69.6%)		27 (39.1%)	42 (60.9%)	
**Left side**	135	36 (26.7%)	99 (73.3%)	0.895	30 (22.2%)	105 (77.8%)	0.087	46 (34.1%)	89 (65.9%)	0.035	30 (22.2%)	105 (77.8%)	0.401	51 (37.8%)	84 (62.2%)	0.031	33 (24.4%)	102 (75.6%)	0.359	38 (28.1%)	97 (71.9%)	0.111
**Tumor size**																						
**< 4.5 cm**	114	24 (21.1%)	90 (78.9%)		25 (21.9%)	89 (78.1%)		43 (37.7%)	71 (62.3%)		25 (21.9%)	89 (78.1%)		49 (43%)	65 (57%)		23 (20.2%)	91 (79.8%)		34 (29.8%)	80 (70.2%)	
**≥ 4.5 cm**	90	31 (34.4%)	59 (65.6%)	0.032	28 (31.1%)	62 (68.9%)		37 (41.1%)	53 (58.9%)	0.622	24 (26.7%)	66 (73.3%)	0.432	39 (43.3%)	51 (56.7%)	0.96	31 (34.4%)	59 (65.6%)	0.022	31 (34.4%)	59 (65.6%)	0.482
**CEA**																						
**< 5 ng/ml**	158	38 (24.1%)	120 (75.9%)		40 (25.3%)	118 (74.7%)		64 (40.5%)	94 (59.5%)		37 (23.4%)	121 (76.6%)		71 (44.9%)	87 (55.1%)		38 (24.1%)	120 (75.9%)		53 (33.5%)	105 (66.5%)	
**≥ 5 ng/ml**	46	17 (37%)	29 (63%)	0.083	13 (28.3%)	33 (71.7%)	0.689	16 (34.8%)	30 (65.2%)	0.484	12 (26.1%)	34 (73.9%)	0.709	17 (37%)	29 (63%)	0.336	16 (34.8%)	30 (65.2%)	0.147	12 (26.1%)	34 (73.9%)	0.339
**T stage**																						
**T1–3**	171	45 (26.3%)	126 (73.7%)		43 (25.1%)	128 (74.9%)		68 (39.8%)	103 (60.2%)		43 (25.1%)	128 (74.9%)		75 (43.9%)	96 (56.1%)		45 (26.3%)	126 (73.7%)		55 (32.2%)	116 (67.8%)	
**T4**	33	10 (30.3%)	23 (69.7%)	0.637	10 (30.3%)	23 (69.7%)	0.536	12 (36.4%)	21 (63.6%)	0.714	6 (18.2%)	27 (81.8%)	0.391	13 (39.4%)	20 (60.6%)	0.635	9 (27.3%)	24 (72.7%)	0.909	10 (30.3%)	23 (69.7%)	0.834
**N stage**																						
**N0**	107	36 (33.6%)	71 (66.4%)		37 (34.6%)	70 (65.4%)		49 (45.8%)	58 (54.2%)		34 (31.8%)	73 (68.2%)		51 (47.7%)	56 (52.3%)		38 (35.5%)	69 (64.5%)		37 (34.6%)	70 (65.4%)	
**N1–3**	97	19 (19.6%)	78 (80.4%)	0.024	16 (16.5%)	81 (83.5%)	0.003	31 (32%)	66 (68%)	0.043	15 (15.5%)	82 (84.5%)	0.006	37 (38.1%)	60 (61.9%)	0.17	16 (16.5%)	81 (83.5%)	0.002	28 (28.9%)	69 (71.1%)	0.382
**TNM stage**																						
**Stage I, II**	110	37 (33.6%)	73 (66.4%)		37 (33.6%)	73 (66.4%)		51 (46.4%)	59 (53.6%)		35 (31.8%)	75 (68.2%)		53 (48.2%)	57 (51.8%)		37 (33.6%)	73 (66.4%)		39 (35.5%)	71 (64.5%)	
**Stage III, IV**	94	18 (19.1%)	76 (80.9%)	0.02	16 (17%)	78 (83%)	0.007	29 (30.9%)	65 (69.1%)	0.024	14 (14.9%)	80 (85.1%)	0.005	35 (37.2%)	59 (62.8%)	0.116	17 (18.1%)	77 (81.9%)	0.012	26 (27.7%)	68 (72.3%)	0.234

Since p53 expression and *TP53* variation showed significant correlations with N stage and TNM stage, we subdivided the p53 aberrant type pattern into missense and nonsense/frameshift types and *TP53* variation into missense and nonsense/frameshift variations and analyzed the correlation between N stage and TNM stage (Tables 6 and 7). For p53 IHC, missense pattern and nonsense/frameshift pattern were significantly associated with higher N stage and TNM stage compared to wild type pattern (Table 6). For *TP53* variation, nonsense/frameshift variation showed significant correlations to higher N stage and TNM stage (Table 7). Missense variations of *TP53* were significantly related with higher TNM stage but not with N stage (Table 7).

**Table 6:** Association between p53 immunohistochemical expression pattern and lymph node stage and TNM stage

Characteristics	p53 IHC (DO-7)		***p***	p53 IHC (DO-7)		***p***	Total	p53 IHC (Bp53–11)		***p***	p53 IHC (Bp53–11)		***p***	p53 IHC (SP5)		***p***	p53 IHC (SP5)		***p***
	Wild type pattern (1 ~ 79%)	Missense pattern (≥80%)		Wild type pattern (1 ~ 79%)	Nonsense/frameshift pattern (0%)			Wild type pattern (1 ~ 49%)	Missense pattern (≥50%)		Wild type pattern (1 ~ 49%)	Nonsense/frameshift pattern (0%)		Wild type pattern (2 ~ 69%)	Missense pattern (≥70%)		Wild type pattern (2 ~ 69%)	Nonsense/frameshift pattern (≤1%)	
**N stage**																			
**N0**	37 (42%)	51 (58%)		37 (66.1%)	19 (33.9%)		90	34 (37.8%)	56 (62.2%)		34 (66.7%)	17 (33.3%)		38 (43.7%)	49 (56.3%)		38 (65.5%)	20 (34.5%)	
**N1–3**	16 (21.1%)	60 (78.9%)	0.004	16 (43.2%)	21 (56.8%)	0.03	75	15 (20%)	60 (80%)	0.013	15 (40.5%)	22 (59.5%)	0.015	16 (22.2%)	56 (77.8%)	0.004	16 (39%)	25 (61%)	0.009
**TNM stage**																			
**Stage I, II**	37 (41.1%)	53 (58.9%)		37 (64.9%)	20 (35.1%)		92	35 (38%)	57 (62%)		35 (66%)	18 (34%)		37 (42%)	51 (58%)		37 (62.7%)	22 (37.3%)	
**Stage III, IV**	16 (21.6%)	58 (78.4%)	0.008	16 (44.4%)	20 (55.6%)	0.052	73	14 (19.2%)	59 (80.8%)	0.008	14 (40%)	21 (60%)	0.016	17 (23.9%)	54 (76.1%)	0.017	17 (42.5%)	23 (57.5%)	0.047

**Table 7:** Association of TP53 variation type with lymph node stage and TNM stage

Characteristics	Total	***TP53*** mutation status		***p***	Total	***TP53*** mutation status		***p***
		Wild type	Missense			Wild type	Nonsense/frameshift	
**N stage**								
**N0**	90	36 (40%)	54 (60%)		53	36 (67.9%)	17 (32.1%)	
**N1–3**	73	19 (26%)	54 (74%)	0.061	43	19 (44.2%)	24 (55.8%)	0.019
**TNM stage**								
**Stage I, II**	92	37 (40.2%)	55 (59.8%)		55	37 (67.3%)	18 (32.7%)	
**Stage III, IV**	71	18 (25.4%)	53 (74.6%)	0.047	41	18 (43.9%)	23 (56.1%)	0.022

### Prognostic impact of immunohistochemical expression of p53 and *TP53* variation in CRC patients

Table 8 shows univariate analysis for OS and RFS of CRC patients. Histologic grade, T stage, N stage, TNM stage, and positive p53 expression (*P* = 0.018) were significantly associated with OS of CRC patients. The CRC patients with positive p53 (DO-7) expression had a 4.35-fold [95% confidence interval (95% CI); 1.29–14.71, *P =* 0.018] increased risk of death. Positive p53(Bp53–11) expression had a 2.79-fold (1.03–7.57, *P =* 0.044) increased risk of death. And positive p53(Bp53–11) expression had a 10.861-fold (1.46–80.78, *P =* 0.02) increased risk of death. Tumor size, T stage, N stage, and TNM stage were significantly associated with the RFS of CRC patients by univariate analysis. However, the variation status of *TP53* gene was not associated with OS or RFS.

**Table 8:** Univariate Cox proportional hazards regression analysis for overall survival and relapse-free survival in colorectal cancer patients

Characteristics	OS		RFS	
	HR (95% CI)	***p***	HR (95% CI)	***p***
**Sex, female (vs. male)**	0.646 (0.263–1.588)	0.341	0.985 (0.473–2.052)	0.968
**Age, y ≥ 50 (vs. < 50)**	1.394 (0.187–10.369)	0.745	1.874 (0.255–13.759)	0.537
**Histologic grade, Poor (vs. Well or Moderate)**	3.324 (1.419–7.784)	0.006	1.211 (0.494–2.964)	0.676
**Site, Left side (vs. Right side)**	0.725 (0.31–1.696)	0.725	1.003 (0.469–2.144)	0.993
**Tumor size, ≥ 4.5 cm (vs. < 4.5 cm)**	1.95 (0.833–4.565)	0.124	2.109 (1.014–4.387)	0.046
**T stage, T4 (vs. T1–3)**	3.262 (1.367–7.779)	0.008	2.695 (1.231–5.899)	0.013
**N stage, N1–3 (vs. N0)**	3.129 (1.224–8)	0.017	4.262 (1.826–9.943)	0.001
**TNM Stage, III or IV (vs. TNM Stage, I or II)**	3.326 (1.301–8.503)	0.012	3.775 (1.679–8.489)	0.001
**CEA, < 5 ng/ml (vs. ≥ 5 ng/ml)**	1.389 (0.542–3.557)	0.493	1.048 (0.425–2.584)	0.919
**p53 (DO-7) IHC, positive, > 55% (vs. negative, ≤55%)**	4.352 (1.288–14.712)	0.018	0.919 (0.446–1.894)	0.818
**p53 (Bp53–11) IHC, positive, > 50% (vs. negative, ≤50%)**	2.79 (1.029–7.566)	0.044	0.854 (0.417–1.752)	0.668
**p53 (SP5) IHC, positive, > 30% (vs. negative, ≤30%)**	10.861 (1.46–80.775)	0.02	0.908 (0.432–1.912)	0.8
***TP53*, Wild type (vs. Variation type)**	1.739 (0.588–5.142)	0.317	1.36 (0.583–3.174)	0.477

**Fig. 2:**
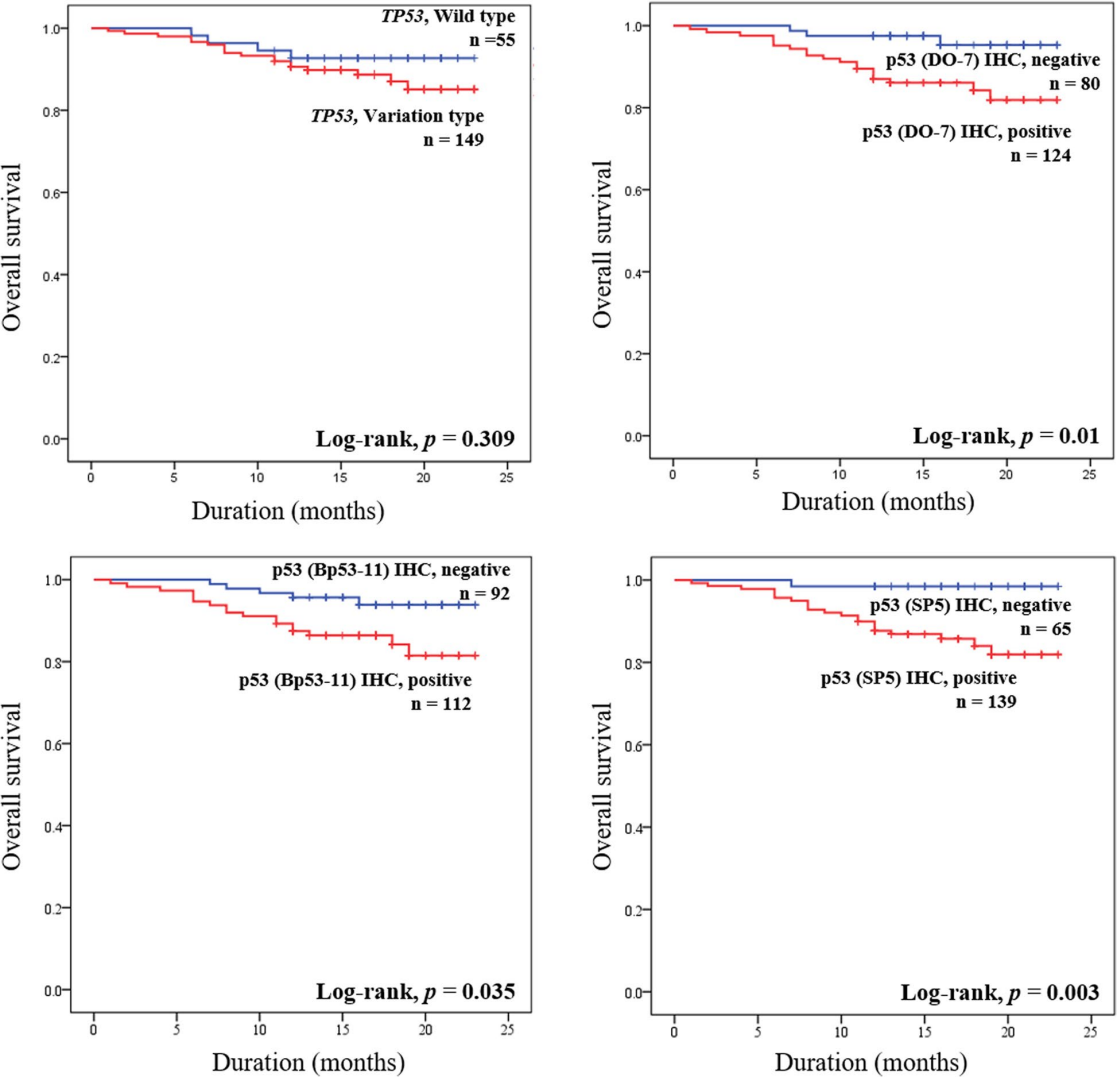
Survival analysis according to variational status of *TP53* and immunohistochemical expression of p53 in colorectal carcinoma patients. Kaplan-Meier survival curves for overall survival of colorectal carcinoma patients according to the immunohistochemical expression of p53 and variational status of *TP53*

Kaplan-Meier survival analysis curves for OS of CRC patients according to *TP53* status or p53 IHC (positive/negative expression) are presented in Fig. 2. Kaplan-Meier survival analysis curves for OS considering the p53 IHC (wild pattern/aberrant pattern) and Kaplan-Meier survival analysis for RFS regarding the p53 IHC and *TP53* status are in supplemental Figs. 1 and 2. For OS, the group with positive expression for p53 had significantly shorter OS than the negative expression group (DO-7: *P* = 0.01, Bp53–11: *P* = 0.035, SP5: *P* = 0.003). The OS of CRC patients with *TP53* variation or aberrant p53 expression pattern did not show a significant difference from the *TP53* wild type or p53 wild type expression pattern. The variation status of *TP53* and p53 expression showed no difference on the RFS of CRC patients.

**Fig. 3:**
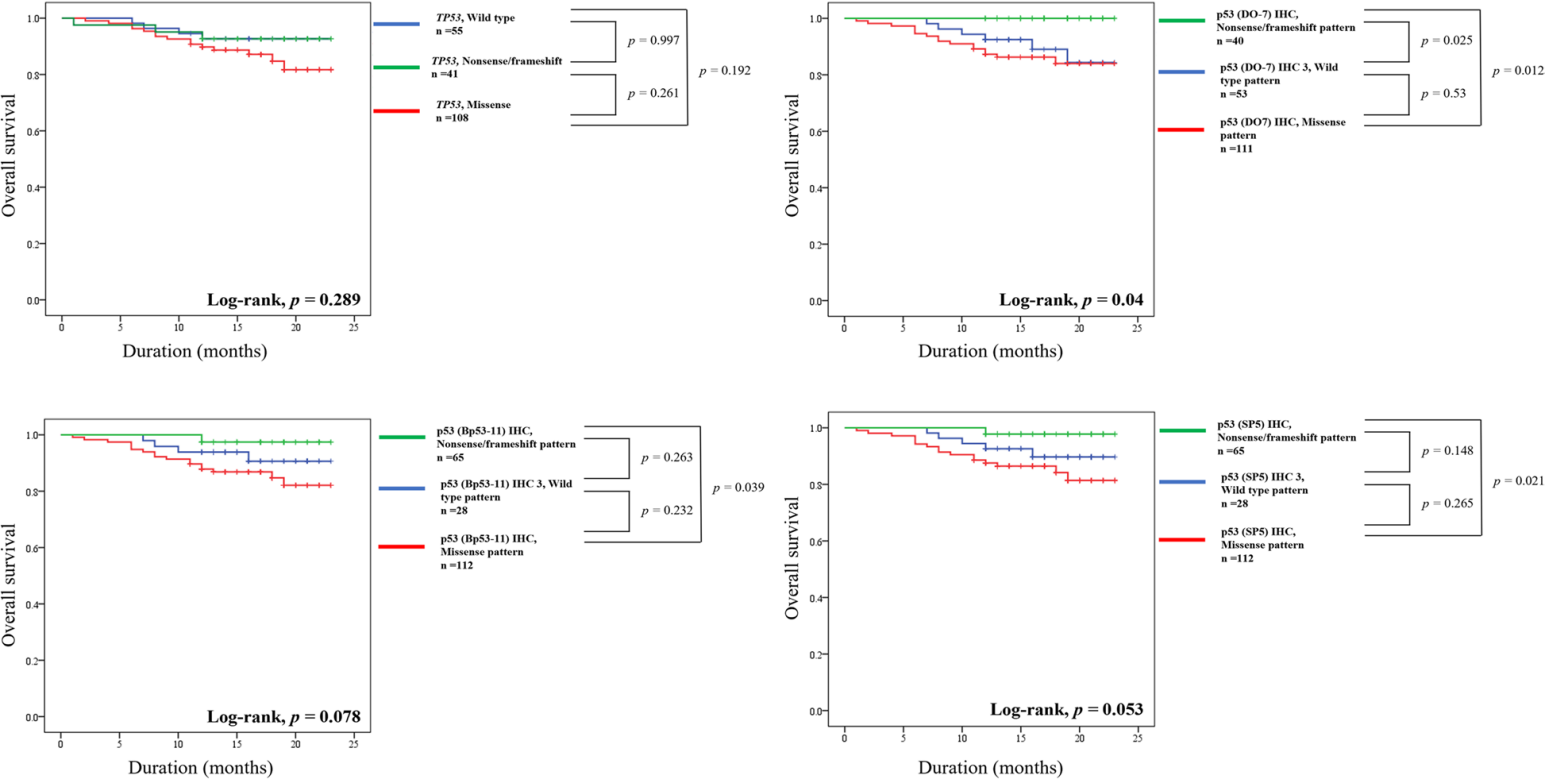
Survival analysis after subclassifying the *TP53* variation and immunohistochemical expression of p53 in colorectal carcinoma patients. Kaplan-Meier survival curves for overall survival after reclassifying the *TP53* variation into nonsense/frameshift and missense variation and aberrant pattern of p53 expression into nonsense/frameshift and missense pattern

In addition, we further divided the p53 aberrant type pattern into missense and nonsense/frameshift type and *TP53* variation into missense and nonsense/frameshift variation and performed Kaplan-Meier analysis for OS of CRC patients (Fig. 3). The p53 (DO-7) expression patterns were significantly associated with OS survival of CRC patients (*P* = 0.04). The CRC patients with nonsense/frameshift pattern of p53 (DO-7) expression showed significantly better prognosis compared to patients with missense or wild type pattern (*P* = 0.012, *P* = 0.025, respectively). Although it was not statistically significant in the other two clones, a similar tendency that nonsense/frameshift pattern of p53 expression showing better OS than wild type pattern or missense pattern was observed. However, there was no significant difference in OS of CRC patients according to type of TP53 variation (Fig. 3).

We performed multivariate analysis for OS and RFS of CRC patients (Table 9). Multivariate analysis included sex, age, histologic grade, site, tumor size, T stage, N stage, and TNM stage. Along with the above-listed variables, multivariate analysis was performed and included positive/negative p53 expression group, and *TP53* variational status in models 1–4. For the OS of CRC patients, histologic grade, TNM stage, and positive/negative p53 expression were independent prognostic factors. For the RFS of CRC patients, only N stage was an independent prognostic factor.

**Table 9:** Multivariate Cox regression analysis for overall survival and relapse-free survival in colorectal cancer patients

Characteristics	OS		RFS	
	HR (95% CI)	***p***	HR (95% CI)	***p***
**Model 1**				
**Histologic grade, Poor (vs. Well or Moderate)**	3.375 (1.431–7.964)	0.005	0.828 (0.328–2.092)	0.69
**N stage, 1–3 (vs. N stage, 0)**	0.61 (0.094–3.982)	0.606	4.262 (1.826–9.943)	0.001
**TNM Stage, III or IV (vs. TNM Stage, I or II)**	2.543 (0.981–6.592)	0.055	1.123 (0.212–5.946)	0.892
**p53 (DO-7) IHC, positive, > 55% (vs. negative, ≤55%)**	4.098 (1.197–14.031)	0.025	0.77 (0.371–1.599)	0.483
**Model 2**				
**Histologic grade, Poor (vs. Well or Moderate)**	3.114 (1.323–7.33)	0.009	0.852 (0.338–2.148)	0.734
**N stage, 1–3 (vs. N stage, 0)**	0.683 (0.101–4.632)	0.696	4.262 (1.826–9.943)	0.001
**TNM Stage, III or IV (vs. TNM Stage, I or II)**	2.76 (1.067–7.142)	2.76	1.113 (0.209–5.917)	0.9
**p53 (Bp53–11) IHC, positive, > 50% (vs. negative, ≤50%)**	2.531 (0.926–6.918)	0.07	0.759 (0.369–1.56)	0.452
**Model 3**				
**Histologic grade, Poor (vs. Well or Moderate)**	3.04 (1.288–7.178)	0.011	0.854 (0.338–2.156)	0.738
**N stage, 1–3 (vs. N stage, 0)**	0.587 (0.081–4.242)	0.598	4.262 (1.826–9.943)	0.001
**TNM Stage, III or IV (vs. TNM Stage, I or II)**	2.674 (1.036–6.903)	0.042	1.125 (0.214–5.928)	0.889
**p53 (SP5) IHC, positive, > 30% (vs. negative, ≤30%)**	9.897 (1.327–73.831)	0.025	0.788 (0.371–1.676)	0.536
**Model 4**				
**Histologic grade, Poor (vs. Well or Moderate)**	3.077 (1.312–7.221)	0.01	0.836 (0.33–2.114)	0.705
**N stage, 1–3 (vs. N stage, 0)**	0.722 (0.083–6.314)	0.769	4.262 (1.826–9.943)	0.001
**TNM Stage, III or IV (vs. TNM Stage, I or II)**	3.122 (1.219–8)	0.018	1.115 (0.211–5.878)	0.898

Model 1 variables: Sex, Age, Histologic grade, Site, Tumor size, T stage, N stage, Stage, CEA, p53 (D07) IHC Negative/Positive

Model 2 variables: Sex, Age, Histologic grade, Site, Tumor size, T stage, N stage, Stage, CEA, p53 (Bp53–11) IHC Negative/Positive

Model 3 variables: Sex, Age, Histologic grade, Site, Tumor size, T stage, N stage, Stage, CEA, p53 (SP5) IHC Negative/Positive

Model 4 variables: Sex, Age, Histologic grade, Site, Tumor size, T stage, N stage, Stage, CEA, *TP53* NGS, Wild type/ Variation type

## Discussion

In the present study, we investigated the immunohistochemical expression of p53 and the variational status of *TP53* by NGS in CRC patients. In the 204 CRC patients, *TP53* variations were detected in 73% of patients (149/204), with 108 (72.5%) patients harboring missense variation and 41 (27.5%) patients with nonsense or frameshift variation. (2) The cutoff value for p53 IHC expression reflecting missense variations was 80%, and the cutoff value for nonsense/frameshift variations was 0%. Subdividing p53 expression into missense (p53 proportion, ≥80%) and nonsense/frameshift (p53 proportion, 0%) patterns showed significant correlation with missense and nonsense/frameshift *TP53* variations, respectively. (3) *TP53* variation and p53 IHC expression showed correlation with poor prognostic factors such as higher N stage and TNM stage. (4) Univariate and multivariate survival analyses indicated positive p53 IHC expression (p53 proportion, > 55%) as an independent factor for poor OS in patients with CRC. (5) Nonsense/frameshift (p53 proportion, 0%) expression pattern of p53 showed a significantly better prognosis than wild type or missense p53 IHC expression pattern.

Currently, immunohistochemical staining for p53 is the tool used most often for evaluating *TP53* variation status. However, after introduction of NGS, sequencing of the *TP53* gene in cancer has been increasing rapidly. Previous reports have demonstrated the correlation between p53 expression and *TP53* variation detection by NGS. In a study on ovarian carcinoma, the authors classified p53 expression into wild type, overexpression, and complete absence [15]. The p53 IHC expression showed good concordance with the variation status of *TP53*. The sensitivity of IHC for detecting gain-of-function variations, loss-of-function variation, and the wild type expression of p53 was 100, 76, and 100%, respectively [15]. The specificity of IHC for detecting gain-of-function variations, loss-of-function variations, and wild type expression of p53 was 95, 100, and 96%, respectively [15]. In gastric cancer, the IHC of p53 expression showed a significant correlation with *TP53* variation detected by NGS. In brain glioma, the sensitivity of p53 IHC for detecting *TP53* variation was 87% [18]. The cut-off point for p53 IHC differs according to organ studied. The cut-off point was 50% in ovarian cancer, 10% in brain glioma, and 50% in gastric cancer. In the present study, we performed ROC curve analysis to set a cut-off point for p53 IHC. The cut-off point was 80 and 1% for missense variation and nonsense/frameshift variation, respectively. On the other hand, there was also a report that the IHC of p53 expression cannot be used to predict *TP53* variations [19]. However, precise validation of the cut-offs related to percent positivity of p53 IHC has been limited in CRC. The reports regarding the correlation between immunohistochemical expression of p53 and *TP53* variation status is summarized in Table 10. To the best of our knowledge, this is the first study to report a correlation between immunohistological expression of p53 and variational status of *TP53* gene in CRC patients. In line with previous reports, our data showed a significant correlation between IHC expression of p53 and variational status of the *TP53* gene. Moreover, we set the cut-off point for IHC of p53 expression by analyzing the ROC curve for variational status of *TP53*. Subclassifying p53 expression into three types (missense, nonsense/frameshift, and wild type) showed better accuracy for detecting *TP53* variations than did subdividing p53 expression into two types, such as positive/negative or wild/aberrant type. Based on these results, if the cut-off point for p53 IHC is appropriately set, the IHC of p53 expression can predict the variational status of *TP53* with high probability.

**Table 10:** Previous reports regarding the association between TP53 variation status and IHC expression of p53

Study	Cancer type	Case number	Clone	Sensitivity	Specificity	Accuracy
Kobel et al., 2016 [15]	Ovarian carcinoma	168	DO-7	96%	100%	98%
Kortekaas et al., 2020	Vulvar carcinoma	59	DO-7	95.3%	100%	96.6%
Singh et al., 2020	Endometrial carcinoma	207	DO-7	90.82%	94.29%	92.26%
Yu et al., 2021	Gastric carcinoma	42	DO-7	100%	77.78%	93.75%
			MX008	95.65%	100%	96.88%
			BP53–12	95.65%	88.89%	93.75%
			SP5	100%	100%	100%
Present study	Colorectal carcinoma	204	DO-7	87.2%	61.8%	80.4%
			Bp53–11	92.6%	69.1%	86.3%
			SP5	91.9%	76.4%	87.7%

Additionally, in predicting *TP53* variation, the sensitivity, specificity, and accuracy of p53 IHC expression show different results depending on the different clones of the p53 antibody. As shown in Table 10, in the study conducted in gastric carcinoma, p53 IHC using SP5 clone predicted the *TP53* variation most accurately. Also, in the present study, it was found that the SP5 clone of the p53 antibody was the best predictor of the *TP53* mutation state. These results suggest that not all p53 antibodies are acceptable in predicting *TP53.* Therefore, when conducting future studies, it is recommended to set the conditions that can most effectively predict the *TP53* variation through the combination of staining conditions and different p53 antibody clones.

The p53 protein, it has been established as a tumor suppressor by extensive studies [20]. Generally, tumor suppressor genes such as *BRCA1*, *RB*, and *APC* lose function through deletions or truncating variations in cancer cells. However, unlike other tumor suppressor genes, the majority of *TP53* variations in cancers is missense variation [21, 22], and most of these occur in the DBD [23]. Our data supported this, showing that 98.1% of the missense variations were located in the DBD. Many studies have confirmed that missense variations can induce tumor progression by a gain-of-function mechanism through regulating proliferation, metastasis, genomic instability, differentiation, metabolism, and immune reactions [23]. In addition, if there is a product missense variation of the *TP53* gene, the mutant protein product is relatively resistant to MDM2-mediated ubiquitination and accumulates in the nucleus of cancer cells, leading to overexpression of p53 [21]. There have been previous reports that p53 overexpression is related to poor survival or progression of CRC in patients [24, 25]. In our study, we investigated the prognosis of CRC patients according to the status of p53 IHC and *TP53* variations.

As with previous reports, our data showed that the CRC patients with negative p53 expression have better OS than CRC patients with positive p53 expression. In addition, multivariate analysis confirmed that positive p53 IHC is an independent poor prognostic factor for CRC patients. However, no other criteria for p53 IHC (wild type pattern/aberrant type pattern) or variational status of *TP53* affected the prognosis of CRC patients. The IHC of p53 expression reveals not only the variational status of *TP53,* but also the post-transcriptional status of the p53 protein. Some reports emphasize the importance of post-translational modification of p53 in tumorigenesis or tumor progression [26, 27]. Our findings and previous reports suggest that the expression status of the p53 protein has a greater impact on the prognosis of CRC patients than does the *TP53* variation itself.

Another interesting finding in our study was that CRC patients with a nonsense/frameshift pattern of DO-7 clone of p53 expression showed significantly better OS than patients with a missense pattern or a wild type pattern of p53 expression. Many studies have been reported on the effect of immunohistochemical expression of p53 on the prognosis of CRC patients. Most of those studies report that CRC patients with p53 overexpression, that is, missense pattern expression, have a poor prognosis. However, there are very limited reports that patients with no or reduced p53 expression have a better prognosis than CRC patients with wild type or missense pattern expression, as in the present study. The p53 protein is actively involved in various DNA damage-response mechanisms [28]. When cells are under stress and experience DNA damage, p53 induces cell-cycle arrest, activates DNA-repair mechanisms, and restores genomic stability [28]. In addition, various DNA-repair systems can be directly activated by the p53 protein [28]. The main adjuvant chemotherapeutic agent for advanced CRC in our institute is oxaliplatin. This agent induces DNA damage by preventing DNA replication. There are numerous reports that mutant p53 (mainly with gain-of-function missense variations) is associated with chemoresistance via various pathways [29–31]. However, we could not find any reports about increased sensitivity to chemotherapy in cells with nonsense/frameshift TP53 variation or absence of p53 expression. In this study, CRC patients without p53 expression had better OS than patients with p53 expression. Based on these results and the results of previous studies indicating that p53 overexpression is related to chemoresistance, we considered the possibility that the group with no p53 expression had better OS through chemosensitivity (or low chemoresistance). However, further studies are needed to determine the chemotherapy susceptibility in cancer cells lacking p53 expression.

Although variations of p53 protein are investigated in the present study, isoforms of p53 protein have been proven to be dysregulated in several human tumors including CRC [32]. Various isoforms of p53 are reported to be involved in development and progression of CRCs [32]. Cell functions affected by the p53 isoforms include apoptosis, autophagy, DNA repair, invasion, angiogenesis, metabolism, and senescence [32]. Moreover, although not much research has been conducted yet, it has been reported that a specific p53 isoform affects the prognosis of CRC. The antibodies used in the present study can capture some isoforms as well (DO7 and Bp53–11 recognize p53β and p53γ; for SP5 the epitope is not determined). However, in this study, the effect of p53 isoforms in CRC patients was not investigated. Therefore, in the future, not only studies on *TP53* variations but also studies on the effect of the various p53 isoforms on the prognosis and treatment of CRC patients might be considered.

In conclusion, our study showed that IHC of p53 expression can predict *TP53* variation status. To predict the prognosis of CRC patients, p53 protein expression is thought to provide more information than the variation itself. In our study, CRC patients without p53 expression had a better prognosis. Further studies are needed to establish the mechanism for differences in OS in CRC patients with or without p53 expression.

## Supplementary Information

**Additional file 1: Supplemental Table 1.** Summary of Coding DNA change and amino acid change of missense variations of *TP53* gene detected in present study. **Supplemental Table 2.** Summary of Coding DNA change and amino acid change of nonsense/frameshift variations of *TP53* gene detected in present study.

**Additional file 2: Fig. S1.** Overall survival analysis according to immunohistochemical expression of p53 IHC (wild/aberrant) in colorectal carcinoma patients. Kaplan-Meier survival curves for overall survival of colorectal carcinoma patients according to the immunohistochemical expression of p53 expression (wild/aberrant).

**Additional file 3: Fig. S2.** Relapse free survival analysis according to variational status of *TP53* and immunohistochemical expression of p53 in colorectal carcinoma patients. Kaplan-Meier survival curves for relapse free survival of colorectal carcinoma patients according to the immunohistochemical expression of p53 and variational status of *TP53*.

## Data Availability

The datasets used and/or analyzed during the current study are available on “https://www.ncbi.nlm.nih.gov/sra” and the accession number is SAMN26687404.
