# Supplementary material for: Clinical significance of p53 protein expression and TP53 variation status in colorectal cancer
Source: BMC Cancer. 2022 Aug 31;22:940. doi: 10.1186/s12885-022-10039-y (PMC9434900; doi:10.1186/s12885-022-10039-y)
Supplement: Supplementary file 1 — Additional file 1: Supplemental Table 1. Summary of Coding DNA change and amino acid change of missense variations of TP53 gene detected in present study. Supplemental Table 2. Summary of Coding DNA change and amino acid change of nonsense/frameshift variations of TP53 gene detected in present study. [file 12885_2022_10039_MOESM1_ESM.docx]

Supplemental Table 1. Summary of Coding DNA change and amino acid change of missense variations of *TP53* gene detected in present study

| cDNA change | Amino acid change | Case number |
| --- | --- | --- |
| c.524G>A | p.Arg175His | 19 |
| c.817C>T | p.Arg273Cys | 13 |
| c.818G>A | p.Arg273His | 7 |
| c.844C>T | p.Arg282Trp | 6 |
| c.730G>A | p.Gly244Ser | 5 |
| c.743G>A | p.Arg248Gln | 5 |
| c.527G>A | p.Cys176Tyr | 4 |
| c.711G>A | p.Met237Ile | 4 |
| c.404G>A | p.Cys135Tyr | 3 |
| c.422G>A | p.Cys141Tyr | 2 |
| c.578A>G | p.His193Arg | 2 |
| c.742C>T | p.Arg248Trp | 2 |
| c.329G>C | p.Arg110Pro | 1 |
| c.423C>G | p.Cys141Trp | 1 |
| c.434T>A | p.Leu145Gln | 1 |
| c.434T>C | p.Leu145Pro | 1 |
| c.451C>T | p.Pro151Ser | 1 |
| c.455C>T | p.Pro152Leu | 1 |
| c.464C>A | p.Thr155Asn | 1 |
| c.475G>C | p.Ala159Pro | 1 |
| c.517G>A | p.Val173Met | 1 |
| c.518T>A | p.Val173Glu | 1 |
| c.527G>T | p.Cys176Phe | 1 |
| c.569C>G | p.Pro190Arg | 1 |
| c.569C>T | p.Pro190Leu | 1 |
| c.578A>C | p.His193Pro | 1 |
| c.612G>C | p.Glu204Asp | 1 |
| c.614A>T | p.Tyr205Phe | 1 |
| c.638G>A | p.Arg213Gln | 1 |
| c.638G>C | p.Arg213Pro | 1 |
| c.641A>G | p.His214Arg | 1 |
| c.658T>C | p.Tyr220His | 1 |
| c.701A>G | p.Tyr234Cys | 1 |
| c.722C>T | p.Ser241Phe | 1 |
| c.725G>C | p.Cys242Ser | 1 |
| c.733G>C | p.Gly245Arg | 1 |
| c.752T>A | p.Ile251Asn | 1 |
| c.761T>A | p.Ile254Asn | 1 |
| c.772G>A | p.Glu258Lys | 1 |
| c.797G>A | p.Gly266Glu | 1 |
| c.810T>G | p.Phe270Leu | 1 |
| c.814G>A | p.Val272Met | 1 |
| c.814G>C | p.Val272Leu | 1 |
| c.818G>T | p.Arg273Leu | 1 |
| c.821T>C | p.Val274Ala | 1 |
| c.832C>T | p.Pro278Ser | 1 |
| c.833C>G | p.Pro278Arg | 1 |
| c.856G>A | p.Glu286Lys | 1 |

Supplemental Table 2. Summary of Coding DNA change and amino acid change of nonsense/frameshift variations of *TP53* gene detected in present study

| cDNA change | Amino acid change | Case number |
| --- | --- | --- |
| c.637C>T | p.Arg213Ter | 5 |
| c.1024C>T | p.Arg342Ter | 4 |
| c.586C>T | p.Arg196Ter | 3 |
| c.880G>T | p.Glu294Ter | 3 |
| c.916C>T | p.Arg306Ter | 3 |
| c.254delC | p.Pro85fs | 2 |
| c.365_366delTG | p.Val122fs | 2 |
| c.1006G>T | p.Glu336Ter | 1 |
| c.1015G>T | p.Glu339Ter | 1 |
| c.1024delC | p.Arg342fs | 1 |
| c.1118delA | p.Lys373fs | 1 |
| c.216_217insC | p.Val73fs | 1 |
| c.216delC | p.Val73fs | 1 |
| c.310C>T | p.Gln104Ter | 1 |
| c.383_384insA | p.Ala129fs | 1 |
| c.403delT | p.Cys135fs | 1 |
| c.493C>T | p.Gln165Ter | 1 |
| c.560delG | p.Gly187fs | 1 |
| c.584delT | p.Ile195fs | 1 |
| c.629_630insA | p.Asn210fs | 1 |
| c.731_734delGCGG | p.Gly244fs | 1 |
| c.743G>A | p.Arg248Gln | 1 |
| c.764_766delTCA | p.Ile255del | 1 |
| c.797_798insG | p.Arg267fs | 1 |
| c.868_869insTCTCC | p.Arg290fs | 1 |
| c.980_981insA | p.Tyr327Ter | 1 |
