# Supplementary figures and images for: Clinical significance of p53 protein expression and TP53 variation status in colorectal cancer
Source: BMC Cancer. 2022 Aug 31;22:940. doi: 10.1186/s12885-022-10039-y (PMC9434900; doi:10.1186/s12885-022-10039-y)

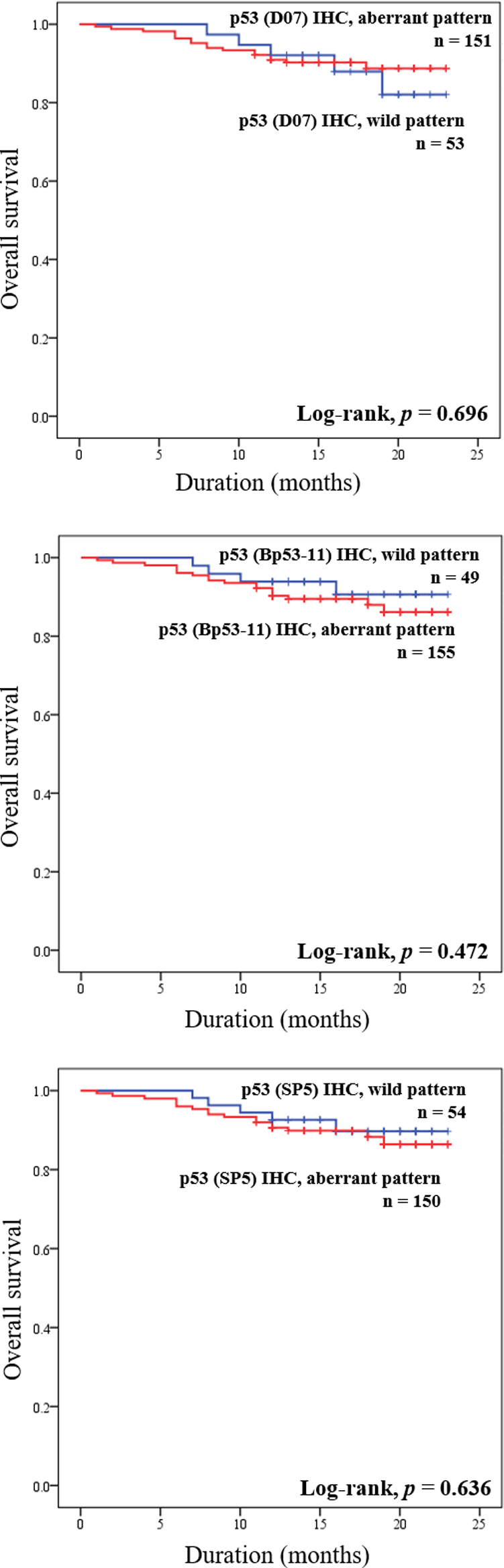

Supplement: Supplementary file 2 — Additional file 2: Fig. S1. Overall survival analysis according to immunohistochemical expression of p53 IHC (wild/aberrant) in colorectal carcinoma patients. Kaplan-Meier survival curves for overall survival of colorectal carcinoma patients according to the immunohistochemical expression of p53 expression (wild/aberrant). [file 12885_2022_10039_MOESM2_ESM.tif]

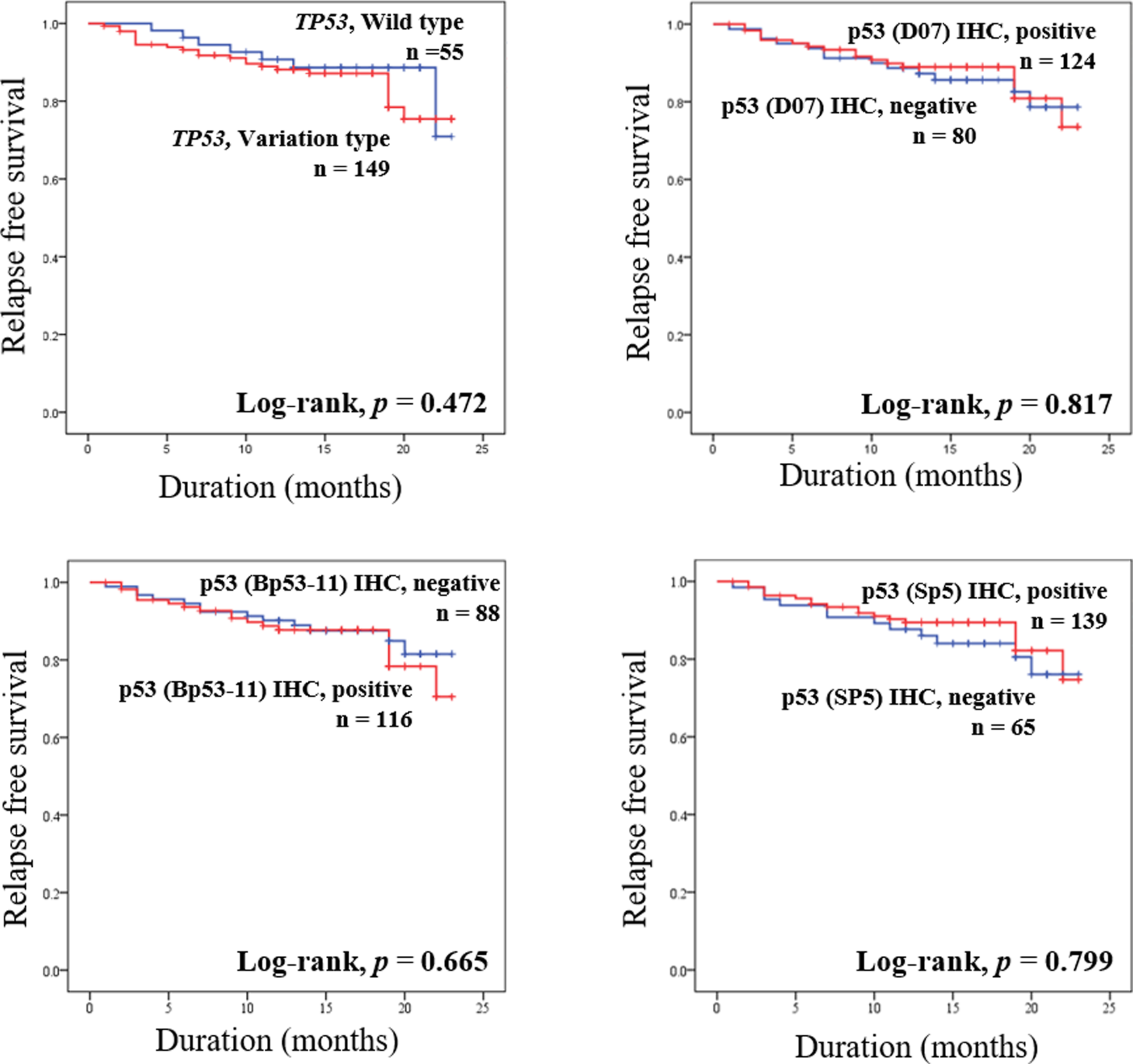

Supplement: Supplementary file 3 — Additional file 3: Fig. S2. Relapse free survival analysis according to variational status of TP53 and immunohistochemical expression of p53 in colorectal carcinoma patients. Kaplan-Meier survival curves for relapse free survival of colorectal carcinoma patients according to the immunohistochemical expression of p53 and variational status of TP53. [file 12885_2022_10039_MOESM3_ESM.tif]
